# Sustainability of health information systems: a three-country qualitative study in southern Africa

**DOI:** 10.1186/s12913-016-1971-8

**Published:** 2017-01-10

**Authors:** Corrina Moucheraud, Amee Schwitters, Chantelle Boudreaux, Denise Giles, Peter H. Kilmarx, Ntolo Ntolo, Zwashe Bangani, Michael E St. Louis, Thomas J Bossert

**Affiliations:** 1 Department of Health Policy and Management, University of California Los Angeles Fielding School of Public Health, 650 Charles Young Drive South, Los Angeles, CA 90095 USA; 2 Centers for Disease Control and Prevention, Maseru, Lesotho; 3 Department of Global Health and Population, Harvard T.H. Chan School of Public Health, Boston, MA USA; 4 Centers for Disease Control and Prevention, Maputo, Mozambique; 5 Centers for Disease Control and Prevention Zimbabwe, Harare, Zimbabwe; 6 Division of Global HIV and TB, Centers for Disease Control and Prevention, Center for Global Health, Atlanta, GA USA; 7 JSI Research and Training Institute, Inc, Lilongwe, Malawi

**Keywords:** Sustainability, PEPFAR, Health information systems, Electronic medical record system, Development assistance

## Abstract

**Background:**

Health information systems are central to strong health systems. They assist with patient and program management, quality improvement, disease surveillance, and strategic use of information. Many donors have worked to improve health information systems, particularly by supporting the introduction of electronic health information systems (EHIS), which are considered more responsive and more efficient than older, paper-based systems. As many donor-driven programs are increasing their focus on country ownership, sustainability of these investments is a key concern. This analysis explores the potential sustainability of EHIS investments in Malawi, Zambia and Zimbabwe, originally supported by the United States President’s Emergency Plan for AIDS Relief (PEPFAR).

**Methods:**

Using a framework based on sustainability theories from the health systems literature, this analysis employs a qualitative case study methodology to highlight factors that may increase the likelihood that donor-supported initiatives will continue after the original support is modified or ends.

**Results:**

Findings highlight commonalities around possible determinants of sustainability. The study found that there is great optimism about the potential for EHIS, but the perceived risks may result in hesitancy to transition completely and parallel use of paper-based systems. Full stakeholder engagement is likely to be crucial for sustainability, as well as integration with other activities within the health system and those funded by development partners. The literature suggests that a sustainable system has clearly-defined goals around which stakeholders can rally, but this has not been achieved in the systems studied. The study also found that technical resource constraints – affecting system usage, maintenance, upgrades and repairs – may limit EHIS sustainability even if these other pillars were addressed.

**Conclusions:**

The sustainability of EHIS faces many challenges, which could be addressed through systems’ technical design, stakeholder coordination, and the building of organizational capacity to maintain and enhance such systems. All of this requires time and attention, but is likely to enhance long-term outcomes.

**Electronic supplementary material:**

The online version of this article (doi:10.1186/s12913-016-1971-8) contains supplementary material, which is available to authorized users.

## Background

Sustainability is a crucial aspect of a program’s life cycle: do activities and benefits continue after original support ends, and what aspects of a program’s design and activities help ensure such longevity? In the realm of global health, donor financing has driven the development and expansion of many initiatives, particularly in the fight against HIV/AIDS [1] – for example, the President’s Emergency Plan for AIDS Relief (PEPFAR), which is “the largest [development fund] by any nation to combat a single disease internationally” [2] and has committed over $65 billion to the HIV/AIDS pandemic since its inception in 2003 [3]. There are numerous other significant sources of funding for HIV/AIDS programs, including other bilateral agencies, the Global Fund to Fight AIDS, Tuberculosis and Malaria, and private donors such as the Bill and Melinda Gates Foundation. However, recent evidence indicates that such donor funding has stagnated [4].

### Health information systems (HIS)

The term health information system(s) (HIS) refers to the collection, storage, management, processing and transmission of information within the health sector [5], and includes things such as: district-level routine information systems, disease surveillance systems, laboratory systems, and/or human resource management information. Electronic HIS can allow for improved timeliness, use, legibility, and data quality, as well as for easier transmission between facilities. Health information systems are critical for a strong health system. They are used to improve disease surveillance, facilitate the strategic use of information, manage patients and programs, and increase service quality through more efficient and efficacious care [6, 7]. Internationally, there are increasing demands for accountability and transparency, so accurate and timely data are important for resource allocation and to monitor and evaluate initiatives’ effectiveness [5, 8].

### Sustainability

Sustainability is the capacity to maintain program services after the end of financial, managerial, and technical assistance from external donors [9]. Table 1 presents factors hypothesized to affect sustainability, as described in the literature, and this was the conceptual framework for this study [10–13]. Program-specific factors include the degree to which project goals are clearly specified and able to show results, the perceived effectiveness of the program in achieving these results, the availability of financing for the program, and the emphasis on training within the program. Relevant organizational factors include the flexibility to modify implementation to meet local needs and conditions, the participatory nature of donor-client and donor-community interactions, the existence of an effective project champion, the extent to which the program is integrated into the host institution and activities, and the institutional strength of the implementing agency. Contextual factors include the presence or absence of concurrent donor projects (either those that compete with the program or those that complement it), the receptivity of the community to participation, and the political, economic and cultural characteristics surrounding the project. Similar determinants have emerged as important for EHIS – for example, political commitment to the system, human resource and infrastructural constraints, physical and socioeconomic environment, alongside global determinants such as donor role, the technological environment, and institutional issues such as the project environment and knowledge management practices [14–17].

**Table 1:** Sustainability framework: determinants of sustainability

Factor	Conditions hypothesized to result in greater sustainability
Program/project-specific factors	
Type/goal(s)	Programs/projects that are better able to demonstrate results, often by being more narrowly focused
Perceived effectiveness	Higher degree
Financing	Ability to secure multiple sources of non-donor financing, particularly from national sources (during or by end of program/project)
Training	Greater emphasis
Organizational factors	
Local-level modifiability	Greater local-level ability to modify implementation to local needs and conditions
Donor-client interactions	Characterized by joint participation/consensus-building
Donor-community interactions	Characterized by joint participation/consensus-building
Project champion	Existing and effective
Integration	Higher degree of integration within host institution, national health authority institution or activities, and/or recipient community needs/priorities
Institutional strength/capacities	Stronger
Contextual factors	
Concurrent projects/donor-supported activities	Fewer similar other programs/projects and/or minimization of competing health problems
Community characteristics	Higher receptivity to participation
Political, economic and cultural characteristics	Socio-political stability, economic stability/growth, higher governmental institutional capacity

This study uses the above-described sustainability framework to inform a case study about the potential sustainability of electronic health information systems (EHIS). The study focuses on work in three sub-Saharan African countries supported by the Centers for Disease Control and Prevention (CDC) under PEPFAR. This article complements a broader emerging literature about the implementation, effectiveness and impact of EHIS in low-resource settings [18–20] including in Latin America [21–23], Asia [24–26] and sub-Saharan Africa [27, 28]. Few studies have emphasized the sustainability of EHIS, particularly with a multi-country comparison and in the context of declining donor support, as is presented here (with some noteworthy exceptions [21, 29]).

## Methods

This was a qualitative study based on interviews with major stakeholders involved in ongoing donor-funded projects for strengthening HIV care through EHIS (as identified through collaborative discussions with in-country experts familiar with the local EHIS) in Malawi, Zambia and Zimbabwe. The interview guide (attached as an Additional file 1) was developed specifically for this study, and used open-ended questions to probe for details about the constructs shown in Table 1. The interview guide was informed by the sustainability framework, and questions were developed to probe different aspects of these constructs.

### Sample selection

Study systems were selected among EHIS projects funded by PEPFAR via CDC, and were chosen to represent systems that had successfully achieved broad national or near-national implementation, and had sufficient tenure of operation to have generated multiple years of sustained exposure among stakeholders. The Zambian and Malawian EHIS are electronic medical record systems, designed and implemented primarily to manage clinical encounters, but also to inform a national data reporting system. The Zimbabwean human resources information system was designed and implemented at the national and provincial levels. A description of the study sites can be found in Table 2.

**Table 2:** Description of study sites and systems

Baobab Health Trust (Baobab) is a non-governmental organization in Malawi that develops and deploys a national electronic medical record system (EMRS). It began its work in 2001, and following an agreement with Luke International in 2012, took the EMRS to national scale. As of 2014, 1.9 million Malawians had been registered in the system. The EMRS targets high-HIV burden facilities and has several modules, including an antiretroviral therapy (ART) module. This supports the clinical management of HIV patients and populates the National HIV Monitoring and Evaluation System. Antenatal care and maternity modules inform the prevention of mother-to-child transmission of HIV (PMTCT) and reproductive health programs. The system also includes an outpatient care module and additional modules for the management of tuberculosis, diabetes and hypertension, for laboratory management, and for national registration and vital statistics. The architecture is open-source and standards-based. Zambia’s SmartCare is also an EMRS system. The system was introduced in 2005 based on more than five years of prior EMRS work in Zambia, and was conceived primarily to improve continuity of care. It remains a patient-oriented system though it has data aggregation capabilities for reporting and analysis at other levels as well. All ART sites in Zambia are required to utilize SmartCare per MOH instructions. The SmartCare system includes multiple clinical modules including ART, voluntary counseling and testing, maternal and child health, and outpatient services. Additional modules are under development. Data from each visit are copied to a local database and to a portable SmartCard that is retained by patients. This dual data collection system allows transfer of an individual’s medical record across facilities. As of November 2013, the program had been deployed to approximately one-third of the country’s 1800 facilities. Zimbabwe’s Human Resources Information System (ZHRIS) was launched in 2009 in collaboration with Emory University. The goals of the system include providing an integrated and interoperable system to routinely produce accurate, high-quality workforce surveillance information for effective decision-making and to advance Zimbabwe’s health leadership capacity in tracking their workforce. The current Zimbabwean National Health Information Strategy calls for a single, central data repository system that integrates routine data on logistics, laboratories, administration, transportation and human resources. The Health Informatics Training and Research Advancement Centre (HITRAC) at the University of Zimbabwe is contracted to develop and deploy ZHRIS, which (as of August 2013) was utilized in all eight provinces and both of the two main cities’ central hospitals in Zimbabwe. Ultimately, the system aims to include real-time data on the training, employment and demographics of the more than 30,000 health personnel working in the country including both those working for the public sector and those employed in the private sector.	

### Data collection

Project researchers from CDC and from Harvard T.H. Chan School of Public Health traveled to the study sites between July and November 2013. All data collectors had training and experience in conducting qualitative research, including data collection and analysis. The team held in-depth discussions with stakeholders from the government, health facilities and implementing partners. Example interviewee types included employees at government ministries, clinical and data/clerical staff at health facilities, and those involved “upstream” in the EHIS (such as software developers, managers, advisors and board members). These respondents were identified by experts in the three countries, and were purposively selected to ensure knowledge of the system and to offer a broad range of experiences with the system. The objective of these interviews was to document features of the EHIS and to identify progress toward country sustainability as per the sustainability framework (i.e., constructs in Table 1). In total, 58 interviews were conducted. Interviews were semi-structured, one-on-one with key informants, based on a standard study protocol, and lasted on average one hour. All participants were given a consent form and were asked to provide oral consent before beginning the interviews. Ethical approval for this study was obtained from CDC, the Harvard T.H. Chan School of Public Health, and host country government offices (Malawi, Zambia and Zimbabwe).

### Data analysis

Interview notes were written by hand. These data were typed up, and were independently analyzed by the interviewers as subsequent interviews were still taking place. This allowed for initial analysis, for any emergent themes to be further evaluated, and served as a method of organizing interview transcripts. Additionally, by beginning to analyze transcripts while data collection is ongoing, researcher(s) can become grounded in the data and additional interviews can be modified to focus on better understanding weak themes and to validate unclear or new ideas brought forward in prior interviews [30].

Patterns were identified from the interview texts, first by inductive analysis for emergent themes, which were then grouped into the constructs of the sustainability framework. This initial coding and sorting of results was done independently by the 3 interviewers [CM, AS, CB] and then this group examined and discussed the preliminary thematic sets of results, particularly focusing on commonalities across all study countries and areas of difference, for example by system type. The narrative synthesis for each construct was written and then checked by revisiting the original interview text to ensure data were not de-contextualized, and that verbatim quotes were interpreted correctly based on their original context. There was also a member checking process, whereby the main results were discussed with the study participants to confirm information received by the interviewers. Other secondary data sources (i.e. program planning documents, progress reports) were also used to triangulate and ensure validity of the results.

## Results

This section details the main study findings as identified across the set of three case studies according to the sustainability framework. Findings are also summarized in Table 3. All findings are presented anonymously as stated in the research protocol and consent form.

**Table 3:** Summary of findings

Factor	Key findings
Program/project-specific factors	
Type/goal(s)	Establishing and communicating goals is important for creating, and gaining buy-in to, a vision; and for measuring success. This was a challenge for all study systems, particularly due to diverse user bases.
Perceived effectiveness	There was optimism regarding the potential for EHIS to ease workload of health staff. But challenges with development and deployment (as may be expected for high-tech systems in low-resource settings) raised concerns that early system glitches may compromise perceptions of reliability, ultimately undermining user buy-in.
Financing	All study systems were reportedly highly dependent on external financing, though all have diversified funding beyond the initial investors. Many respondents perceived a high cost to maintain such a system, and a relatively low priority for the EHIS within national budgets. The burden of “donor dependence” and possible “mission creep” were also discussed.
Training	Training has been a major component of all three systems. Respondents noted that full integration of EHIS into the health system may require widespread and appropriate training, including for all levels of managers to increase data use, and technical training to ensure maintenance of these complex systems.
Organizational factors	
Local-level modifiability	System adaptability was associated with a number of implementation challenges. A centrally-designed system was criticized for its limited utility on-site. Flexible systems struggled to keep pace with users’ development requests, and the lack of standardization could slow software development and deployment.
Donor-client interactions	Communication around EHIS support was reportedly positive, but implementing partners (and other development partners) frequently expressed a desire for more feedback, especially regarding organizational performance.
Donor-community interactions	The community of system users generally perceived all three EHIS as Ministry-led activities, a strong reflection of institutionalization. Stakeholders noted a challenge around timing: if system users are engaged before the EHIS is robust, this could lead to disappointment and discontinuation of use.
Project champion	The presence of a project champion was very often perceived to be important for sustainability. It was noted that championship could “trickle up” (from facility-based users) as well as “trickle down” (from central Ministries).
Integration	All study countries had existing health data collection information systems, and all faced challenges in building synergies with the EHIS rather than duplication. The ongoing presence of (duplicative) paper-based data collection was an important frustration for system users in all three countries.
Institutional strength/capacities	The importance of capacity was highlighted across many levels: in developing and maintaining the EHIS; in system implementation and scale-up; and in building momentum for EHIS as a national priority. Users’ computer literacy and technology infrastructure have impacted system (hardware and software) design and deployment.
Contextual factors	
Concurrent projects/donor-supported activities	EHIS sustainability is strengthened by complementary activities, such as training on data use. Competing EHIS can undermine system standardization; in one example, failure to reach ex-ante consensus on national EHIS needs led to disagreements and a group who lobbied for introduction of an alternative EHIS.
Community characteristics	Stakeholders widely expressed enthusiasm about the systems’ potential, and excitement to be a leader in new technology. Enthusiasm of downstream users (at the health facility level) depended on the system’s potential to lessen workload and reduce reporting requirements.
Political, economic and cultural characteristics	All three countries’ health systems are largely dependent on public sector care delivery, and on financing from external donor sources. Importantly, worldwide trends in computing may lower local costs and increase the inevitability of introducing EHIS.

### Program-/project-specific factors

#### Type/goals

Consensus around program vision was found to be a particular challenge for the two systems that involve a range of activities and a diverse set of users. For these EHIS, few users were able to articulate the full breadth of the systems’ stated goals and metrics, and stakeholders frequently perceived only those system goals that coincided with their own work duties. For example, service providers emphasized clinical applications, administrators focused on planning and resource allocation, and those involved in monitoring and evaluation prioritized indicator reporting. Meanwhile, many respondents with a more global understanding of the system indicated that it was unclear whose perceptions of effectiveness matter most, while others highlighted the complexity of responding to many goals with a single system. One respondent noted that “if we do not concentrate on what we have right now… we will spread ourselves so thin that we will have no effect.” These two EHIS were also perceived to lack explicit methods to evaluate progress towards program objectives. Combined with the compartmentalized nature of Ministries of Health, which tended to isolate health information within a single unit, respondents noted that the lack of monitored progress exacerbated the perception that systems’ roles and impacts were limited.

#### Perceived effectiveness

The importance of perceived effectiveness was a universal finding in the case studies. As one respondent noted, there is a “limit of faith” for the EHIS, and respondents in each of the three countries expressed concerns that system use would stop if users did not see tangible benefits. However, there was an apparent recognition that even promising systems may face early setbacks and challenges; this is partly the nature of information technology deployment and software development. Respondents clearly vocalized frustration with the systems that were being replaced – paper-based information systems were viewed as antiquated and burdensome – and expressed optimism that each of the three systems has potential to significantly improve management and delivery of health services. However, concerns regarding EHIS reliability led to the maintenance of parallel paper systems well after EHIS introduction. Maintenance of duplicate systems was perceived to increase workload and ultimately limit the perceived and actual effectiveness of the EHIS. Reflecting on setbacks that had resulted in the failure of previous systems, one respondent noted that similar issues continue to frustrate current efforts. He succinctly captured how optimism combines with concern, “it can change the way that … decisions are being made” in the country, but added “[I] hope someone will not tire [from these problems] along the way.”

#### Financing

Nearly all respondents acknowledged concerns about financing. Each of the systems relies (to a differing extent) on contributions from external sources, and there was a near-universal perception that the systems would not exist without substantial external financing. While external financing presents opportunities, this reliance was seen to carry risks. Donor-sponsorship comes with deliverables and some respondents voiced concerns that donors use their influence on the content and deployment of EHIS to meet their own priorities and deadlines. These factors may limit perceived effectiveness as well as championship and integration (see below). On the other hand, dependable donor funding was seen as a way to lessen pressure around securing additional funding, thereby enabling focus on the EHIS primary objectives. In one setting where the EHIS was seeking diversification of funding outside PEPFAR, stakeholders were nervous about the possible “mission creep” and the reporting requirements that would likely accompany any additional financing.

The study systems include notable examples of low-cost technologies that innovate around resource constraints, including alternative approaches to power and network connectivity issues (e.g., locally-adapted low-power screens). However, many stakeholders alluded to the high cost of even relatively inexpensive EHIS: “how could [country] justify paying for a computer system if we can’t even pay for medicines?” Another respondent from a different study country stated, “if donor funding were to leave I don’t see the government able to pay that. They can’t even afford our own salaries. Without you people, we are done.” Nonetheless, there is evidence of increasing national financial commitment to operating the systems in all three cases. Examples include paying for consumables such as printer ink, contributing to supporting infrastructure for the EHIS, and introducing a new cadre of information technology officers to assist in troubleshooting and maintenance of EHIS.

#### Training

Respondents perceived training to bridge multiple aspects of sustainability. For example, institutional capacity is built through trainings. Also, large-scale training efforts are seen to increase exposure and buy-in to an EHIS, which may foster a champion and ease the system’s integration into core workplace activities. Perceived effectiveness may be strengthened if users have knowledge and tools to make system information useful to them. Some respondents noted the potential gains of training a variety of partners.

All three EHIS projects have conducted extensive training activities. Different models were described, from formal certification procedures to on-the-job peer-based trainings. Respondents viewed training as essential to building and bolstering the EHIS program, but reported challenges including limited baseline computer literacy, difficulty meeting the need for training (due both to rapid system scale-up and high turnover of users), the costs associated with travel and per diems for trainers and participants, the opportunity cost of lost clinical time, and possible knowledge dilution within peer-training models. Despite these challenges, the importance of such capacity-building was mentioned repeatedly. Trainings reportedly help build computer competency and comfort. It was reported that many trainees had never used a computer before – and in one illustrative example, a respondent spoke about using a computer for the first time during EHIS training, and her subsequent pride about then training peers on the system.

However, ongoing capacity gaps were reported by users of each of the three systems. In particular, stakeholders at all levels described an ongoing need for training that expands technical knowledge. Critically, all three study systems are run by implementing organizations outside of the government and these organizations remain the first responder for most troubleshooting. This was seem as perhaps diminishing system confidence and sustainability, particularly for users in remote areas where technical support may be slow to arrive. However, successful efforts to address this issue were identified: for example, one system trains “super-users” to locally resolve some technical issues, to decrease system downtime. In two of the study countries, the governments have made administrative commitments to taking on some technical support responsibilities – but in both instances capacity gaps remain, and the necessary financial and technical resources are not yet deployed.

It is essential to train those who will be entering data into the EHIS, but multiple respondents commented on the importance of also training managers and Ministry administrators about data use and quality because, to quote one respondent, “appreciation [of the system] is lacking among the senior management.”

### Organizational factors

#### Local-level modifiability

Locally-modified EHIS – developed to meet local needs and with early and extensive engagement of in-country partners – may find greater traction and increased sustainability. To the extent that a consensus is reached early and effectively, this adaptation period was seen as helping to ensure the standardization of information and harmonization across stakeholders. To quote a respondent, “[the EHIS] is sustainable but only if we listen to the users first… Listen to them and then engineer.” A respondent in another country noted that there are “smart guys doing the software development, but they need provider input too.”

In all of the countries visited, stakeholders provided system and software feedback, although there were differences in the speed and uptake of this feedback. Notably, software deployment mechanisms varied – and in the countries where upgrades were done in-person by employees of the managing EHIS organization (which is time- and resource-consuming, relative to web-based downloads of the software which can be done at each site, for example, by trained “super-users” as described above) users expressed more frustration with the speed at which they saw system improvements.

#### Donor-client interactions

Importantly, some respondents saw the newly-introduced EHIS as a donor priority at odds with local needs. To quote one respondent, “we are always skeptical about these sorts of projects… Programs come and distort the system, then we have to start over. . . But we try to be optimistic.” In another country, a program technician noted, “Right now, we worry that, without us going on and demanding the report, [the EHIS] won’t be there.” This overriding skepticism could reportedly undermine the transition away from locally-developed, long-standing paper-based information systems.

Despite these challenges, all sites reported strong working relationships with the donor agencies that support the EHIS, and participatory, collaborative efforts for planning and reporting. Such interactions also sometimes include donor membership on EHIS-relevant working groups and committees within the Ministry of Health. However, many stakeholders mentioned a desire for greater feedback from donors about organizational performance of the EHIS, emphasizing again the importance of a clear focus and measurable outcomes.

#### Donor-community interactions

Many respondents noted weaker ties for donor-community interactions, where the “community” of an EHIS comprises its users (health workers, data clerks, and managers). In one study country, stakeholders perceived that end users saw the EHIS “as a black box” and might wonder “what’s the government up to?” Few respondents clearly acknowledged the donor source or donor role in the EHIS, and instead noted activities and policies as initiated by the Ministry of Health. These findings may reflect efforts to build buy-in and to institutionalize the systems within facility operations.

Identifying the ideal moment to engage system users was highlighted as a particular challenge in the study countries. Respondents felt that reaching out too soon led to unrealistic expectations – and if early iterations of the system could not meet these expectations, frustration and decreased buy-in. Several respondents recalled instances of systems that had strong early engagement but ultimately failed due to delays in procurement or in program updates. One respondent noted a history of challenges with system use, remarking that “we still have low confidence in the system… even where it is working well, people have doubts.” In another country, the current EHIS is a new iteration of earlier software that, in the words of a respondent, “died a natural death for lack of funds.” This early experience had a lasting impression, and the respondent now requires paper-based data collection in addition to the electronic entries, “in case this thing crashes.”

#### Project champions

The EHIS in this study faced challenges with project champions. Respondents noted the importance of high-level engagement and commitment to new systems, especially ones as complex as EHIS—but respondents in all three countries expressed concern that such engagement is lacking. Respondents in one country noted “in meetings, you feel like the government is saying all the right things, but they are still not taking over,” and only with government ownership will they “feel totally responsible if the system fails.”

Championing may also occur “bottom-up.” In one instance the clinical staff at a large urban hospital helped co-develop the EHIS to respond to their needs, and the resulting system was thoroughly integrated to their continuum of care, resulting in widespread use by service providers. A respondent in a different study country noted that “facilities are the best champions. Once they commit, they work to make the system their own, to make it work for them, and to push it forward.”

#### Integration

The need for smooth integration within the broader health information system was highlighted often. In particular, stakeholders expressed a desire to complement ongoing data collection activities across levels of the health system, from the central Ministry of Health to facilities. Many countries have existing information systems for collecting health data, and respondents noted the need to build synergistic rather than duplicative systems. They saw parallel systems as adding workload, reinforcing multiple record-keeping mechanisms (e.g. paper- and computer-based), and detracting from the perceived efficacy of the EHIS.

In all three countries respondents wanted the systems to incorporate information from, and inform decision-making across, different departments. In one study country, facilities using the system throughout patient interactions reported decreased workload and increased care quality, thus increasing buy-in and fostering sustainability. In this same country other sites have not well-integrated the EHIS – one respondent noted that “at the talking level, there is talk” about integration but little actual progress to date. When integration is not achieved, the software essentially operates as a standalone data collection system entirely outside the care experience.

However, integration carries risks. This was highlighted by the many stakeholders who were concerned with spillover effects from potential discontinuation of an EHIS that had become fully embedded in the daily workflow. According to one respondent (at a facility where paper records were not well-maintained) the EHIS was embedded to the point that a “sudden stop would be a disaster.” Integration across systems was similarly seen as risky: if broader information systems are dependent on the EHIS, then a glitch in one component could have far-reaching effects on the country’s capacity to aggregate sector-wide data. In the words of one respondent, “if we keep [this EHIS] completely separate [from other reporting systems] then it can be easily divorced.”

#### Institutional strength/capacities

Almost all study stakeholders emphasized the crucial role of institutional capacity of the relevant partner institutions. This need for capacity was highlighted across a spectrum of activities including developing the software, adopting and implementing the system at the facility level, and setting EHIS as a national priority. Computer literacy and comfort are essential for the successful deployment of EHIS. Respondents perceived that simpler and less technically-complex systems may face fewer capacity constraints, although many noted that this latter issue was rapidly losing importance due to overall increases in computer literacy. Software development and technical support were seen as more substantial challenges to systems’ sustainability. Concerns linked to these issues were raised in each of the three countries visited. The high cost and relative scarcity of strong software and information technology developers and support was a universal concern, and countries were forced to rely on donor support to finance these critical inputs to EHIS development and maintenance.

### Contextual factors

#### Concurrent projects/donor-supported activities

EHIS support did not occur in isolation in any of the countries visited. In all cases activities were strengthened by support for complementary projects – including broader EHIS activities as well as training on the use of data for decision-making. Importantly, the study EHIS was the nationally agreed-upon standard system in all three countries. This carried both opportunities and challenges. The lack of competing activities enabled easier standardization and harmonization across sites. This also eased portability of system knowledge and skills when trained users change job locations within the health system. However, early adoption of a single system reportedly introduced challenges in meeting local needs when sufficient consensus was not reached ex-ante. For example, when the primary system in one country failed to provide all data needed by the system’s many stakeholders, individual organizations began to lobby for the introduction of alternative electronic medical record systems. Notably, despite the official emphasis on a single system, the study team encountered a number of alternative data and information endeavors in this country, sometimes sponsored by key implementing partners of the study EHIS. While these other activities may have increased the availability of hardware and general computer literacy, as well as fostered a broader environment of data collection and use, there were cited instances of other software solutions “competing” with the study EHIS. This competition introduces additional challenges, notably the increased workload for health workers faced with multiple systems and weakened data compatibility.

#### Community characteristics

The community of system users universally expressed receptivity to participation, often greeting the new EHIS with great excitement. EHIS were perceived to be a tangible tool for health system strengthening at the facility level and beyond. However, as noted above, this early excitement carries risk. Some stakeholders discussed facing increased pressure to add new sites, risking deployment of the EHIS at a faster rate than could reasonably be supported within current organizational structures and budgets. Additionally, some respondents emphasized the need to achieve buy-in at all levels, noting cases in which managers were excited about the EHIS but staff were not equally enthusiastic about adding the system to their workload. The new EHIS would create more work for already-overtaxed health workers since most sites continued to maintain paper-based records. It was mentioned several times that any users with initial hesitation around the system had them allayed once they saw tangible benefits of EHIS use – and when these benefits were not obvious, system use waned.

#### Political, economic and cultural characteristics

The three study sites have differing political, economic, and cultural characteristics. Nonetheless there are some commonalities in their health systems that may affect EHIS sustainability. First, the majority of care is delivered through the public sector so the Ministry of Health has a large role in planning and delivering services. Second, all are highly reliant on donor funding, particularly for the HIV sector. This was acknowledged by many respondents with challenges as discussed earlier. Third, the region has a health workforce shortage. Many stakeholders cited this as a challenge in EHIS implementation.

There were discussions about how broad contextual factors are likely to positively impact sustainability of an EHIS. Importantly, many stakeholders noted recent shifts in information technology. Familiarity with software has been cited as generally enabling EHIS implementation [18] and computer literacy has been rapidly increasing in southern Africa. There are expanded training opportunities at the university and certificate levels, and greater computer literacy and comfort in general. In two instances local clinical education programs added specialized training on the EHIS to the standard curriculum. Respondents also noted a broader push for electronic recordkeeping across all sectors. Attitudes towards data were also changing, with increasing focus on how data can be used to inform decision-making, and on the importance of protecting information and using it appropriately.

## Discussion

This study aimed to better understand how to build a promising environment for EHIS investments by exploring constructs from health systems sustainability frameworks. Many studies of health program sustainability have looked at traditional clinical or public health services, but EHIS have unique characteristics – from technical complexity to interactions with broader societal and technological trends – that may differentially affect program sustainability, especially in resource-poor settings.

These case studies highlight the importance of aligning perspectives across partners and across levels of the health system. Such harmonization will improve EHIS sustainability by aligning incentives and setting manageable expectations while more robustly integrating EHIS activities into the health system. Agreement on system vision and goals, and on how to measure these, was frequently noted by participants to be a crucial step in introducing a successful and sustainable EHIS. Accordingly, clarity and concordance on goals also enabled championing whether top-down or bottom-up; this has been found to be important in other instances of EHIS implementation as well [27].

Attaining such universal agreement on the mission and role of an EHIS may be an important goal, but it is a difficult one to achieve [19]. Different partners may enable this in a variety of ways. Donors can encourage participatory and consensus-building activities to set comprehensive project goals and metrics. Governments should also take an active role in such processes, engaging stakeholders from different sectors, including clinical areas, monitoring and evaluation, and planning departments. Finally, EHIS organizations can strive for transparent strategic planning and monitoring processes. Other studies have cited institutionalizing EHIS and other information systems as an important aspect of sustainability [29], citing the risk of such projects remaining in “pilot mode” if they do not have a long-term, multi-partner implementation commitment [21].

System users can play a central role in project design, and their inclusion may help improve sustainability. A main objective of EHIS is to reduce burden on health workers. The additional burden of parallel paper- and computer-based systems can be, and has been, a key challenge to the introduction and implementation of these systems in these three countries, and has been cited elsewhere in the literature [24, 28]. Managers and policymakers in all three countries emphasized the systems’ potential for simplifying and accelerating data collection. However, those tasked with operating the systems frequently expressed frustration that the dual management of paper and electronic records resulted in duplication, rather than elimination, of efforts. Frustrations were especially high among health workers who had been promised that EHIS would streamline their workflow. Abandoning paper in favor of electronic recordkeeping requires a leap of faith, and EHIS implementers should request such a switch only when the system can reasonably handle this demand. Nonetheless, such a transition should be a near-term goal even if it can only be achieved incrementally by specific modules within the software or in only some geographic areas of a country. Respondents from each of the three countries emphasized the fact that system users must perceive tangible benefits if we expect them to use, let alone rely upon, the EHIS. The sooner this can happen, the more reasonable it is to move the health system towards computer-only information systems. Perceived usefulness has emerged in the broader literature as an important determinant of EHIS success [25].

The dynamic nature of the health workforce also emerged as an important issue. The low level of computer literacy within the health workforce is an important constraint to fully effective EHIS deployment. High staff turnover exacerbates this challenge, resulting in constant pressure for training on new EHIS systems. EHIS projects can mitigate the effects of this on system sustainability by giving health workers portable skills, for example, via improved pre-service trainings and standardized software and use protocols throughout the health system. This would reduce the need for constant re-training as health workers move around within the system. It is also crucial to increase public sector salaries to avoid losing trained staff (from EHIS developers to downstream health workers) to private sector employers also in need of their skills. Computer literacy is likely a waning challenge as more youth enter the workforce already familiar with computing, and EHIS trainings may need to adapt their focus from basic skills-building to improved use of technology for providing efficient and high-quality care. EHIS training should be viewed as having an important spillover effect in general increased computer comfort. Human resources has also been cited as a critical factor for successful EHIS implementation and sustainability in other studies from low-resource settings [18, 20, 22].

Lastly and particularly notably, this study highlights the importance of understanding sustainability as a concept that reaches beyond financial stability. There are many other essential determinants of programs’ likely perpetuation and donors should invest in activities that support these additional pillars of sustainability.

This assessment has some limitations. First, the three selected cases are a small sample, and were purposively selected for their successful broad national or near-national EHIS implementation and adoption. Therefore, these cases likely reflect stronger, more effective implementations of EHIS projects than average. Conversely, this arguably makes the experiences of this set of selected systems more important than more limited implementations of EHIS projects, given the high stakes for the health system if such widely-implemented systems are not sustained. All were funded by the same organization (PEPFAR). This eliminates variability in donor characteristics, which might have affected outcomes, but may limit the external validity and generalizability of the results. Additionally this assessment interviewed a limited number of stakeholders per country. It should be noted, however, that interview responses were highly consistent across countries. As a qualitative exercise, there is no statistical test on the internal validity of these results and no way to control for omitted variables or other sources of bias. The diverse study team participated in different ways throughout the research process (including study design, data collection and analysis) which may have helped mitigate this problem, but it cannot be eliminated completely. Lastly, these systems are undergoing many changes and this research captured only a “snapshot” of these efforts. An ideal study design would include future assessments including after a change or termination in funding, to empirically evaluate the predictions generated here.

Our goal was not to assess the merit of EHIS investments, which is a separate and important discussion that should be informed by national priorities as well as data from impact evaluations and cost-effectiveness analyses [31, 32]. These have been acknowledged to be largely lacking in the literature around EHIS [33]. We encourage further study on these topics, as well as on other important EHIS-related issues such as security, data confidentiality, and the appropriateness of EHIS investments by donors given many competing program priorities and the potential for misalignment of needs among national and international stakeholders.

## Conclusions

A broad range of stakeholders confirmed the importance of a number of sustainability determinants, both in guided interview questions corresponding to the framework, and in open-ended questions to elicit their own unprompted perceptions of critical determinants. These findings underscore the importance of creating an enabling environment for program sustainability, including by fostering communication between stakeholders for aligning perspectives and agreeing on a system’s goals, engaging users in the design and implementation process, and taking a broad view of sustainability that looks beyond financial dimensions to other important determinants. This will be critical for donor-led investments such as EHIS in low-resource settings. Achieving sustainability is a resource-intense endeavor, but will be necessary to ensure the long-term success of these programs and to see improved health outcomes.

## Additional file

Additional file 1: Health information system sustainability interview questions. (DOCX 18 kb)

## Abbreviations

ART: Antiretroviral therapy
CDC: Centers for Disease Control and Prevention
EHIS: Electronic health information systems
EMRS: Electronic medical record system
PEPFAR: President’s Emergency Plan for AIDS Relief
PMTCT: Prevention of mother to child transmission

## References

[1] Ravishankar N, Gubbins P, Cooley RJ, Leach-Kemon K, Michaud CM, Jamison DT, Murray CJL (2009). Financing of global health: tracking development assistance for health from 1990 to 2007. Lancet.

[2] Office of the US Global AIDS Coordinator, US Department of State (2010). The U.S. President’s emergency plan for AIDS relief: 2009. Annual report to congress on PEPFAR program results.

[3] PEPFAR Funding. [http://www.pepfar.gov/documents/organization/252516.pdf]. Accessed 29 Dec 2016.

[4] Efficient and Sustainable HIV Responses: Case studies on country progress. [http://www.unaids.org/sites/default/files/media_asset/JC2450_case-studies-country-progress_en_0.pdf]. Accessed 29 Dec 2016.

[5] World Health Organization, Health Metrics Network (2011). Country health information systems: a review of the current situation and trends.

[6] Design and implementation of health information systems. [http://apps.who.int/iris/bitstream/10665/42289/1/9241561998.pdf]. Accessed 29 Dec 2016.

[7] Fryatt R (2014). Investing in health. Lancet.

[8] Aqil A, Lippeveld T, Hozumi D (2009). PRISM framework: a paradigm shift for designing, strengthening and evaluating routine health information systems. Health Policy Plan.

[9] Shediac-Rizkallah MC, Bone LR (1998). Planning for the sustainability of community-based health programs: conceptual frameworks and future directions for research, practice and policy. Health Educ Res.

[10] Bossert TJ (1990). Can they get along without us? Sustainability of donor-supported health projects in Central America and Africa. Soc Sci Med.

[11] Stirman SW, Kimberly J, Cook N, Calloway A, Castro F, Charns M (2012). The sustainability of new programs and innovations: a review of the empirical literature and recommendations for future research. Implement Sci.

[12] Gruen RL, Elliott JH, Nolan ML, Lawton PD, Parkhill A, McLaren CJ, Lavis JN (2008). Sustainability science: an integrated approach for health-programme planning. Lancet.

[13] Scheirer MA (2005). Is sustainability possible? A review and commentary on empirical studies of program sustainability. Am J Eval.

[14] Ludwick DA, Doucette J (2009). Adopting electronic medical records in primary care: lessons learned from health information systems implementation experience in seven countries. Int J Med Inform.

[15] Gordon AN, Hinson RE (2007). Towards a sustainable framework for computer based health information systems (CHIS) for least developed countries (LDCs). Int J Health Care Qual Assur.

[16] Garde S, Hovenga EJ, Gränz J, Foozonkhah S, Heard S (2007). Managing archetypes for sustainable and semantically interoperable electronic health records. Electron J Health Inform.

[17] Luna D, Almerares A, Mayan JC, González Bernaldo de Quirós F, Otero C (2014). Health informatics in developing countries: going beyond pilot practices to sustainable implementations: a review of the current challenges. Healthcare Inform Res.

[18] Fritz F, Tilahun B, Dugas M (2015). Success criteria for electronic medical record implementations in low-resource settings: a systematic review. J Am Med Inform Assoc.

[19] Tamrat T, Kachnowski S (2012). Special delivery: an analysis of mHealth in maternal and newborn health programs and their outcomes around the world. Matern Child Health J.

[20] Oluoch T, Santas X, Kwaro D, Were M, Biondich P, Bailey C, Abu-Hanna A, de Keizer N (2012). The effect of electronic medical record-based clinical decision support on HIV care in resource-constrained settings: a systematic review. Int J Med Inform.

[21] Farach N, Faba G, Julian S, Mejía F, Cabieses B, D’Agostino M, Cortinois AA (2015). Stories from the field: the use of information and communication technologies to address the health needs of underserved populations in Latin America and the Caribbean. JMIR Public Health and Surveill.

[22] Fraser HS, Thomas D, Tomaylla J, Garcia N, Lecca L, Murray M, Becerra MC (2012). Adaptation of a web-based, open source electronic medical record system platform to support a large study of tuberculosis epidemiology. BMC Med Inform Decis Mak.

[23] Graven M, Allen P, Smith I, MacDonald NE (2013). Decline in mortality with the Belize integrated patient-centred country wide health information system (BHIS) with embedded program management. Int J Med Inform.

[24] Gera R, Muthusamy N, Bahulekar A, Sharma A, Singh P, Sekhar A, Singh V (2015). An in-depth assessment of India’s Mother and Child Tracking System (MCTS) in Rajasthan and Uttar Pradesh. BMC Health Serv Res.

[25] Wang J-Y, Ho H-Y, Chen J-D, Chai S, Tai C-J, Chen Y-F (2015). Attitudes toward inter-hospital electronic patient record exchange: discrepancies among physicians, medical record staff, and patients. BMC Health Serv Res.

[26] Ismail NI, Abdullah NH, Shamsudin A, Ariffin NAN (2013). Implementation differences of hospital information system (HIS) in Malaysian public hospitals. Technology.

[27] Cline GB, Luiz JM (2013). Information technology systems in public sector health facilities in developing countries: the case of South Africa. BMC Med Inform Decis Mak.

[28] Tilahun B, Fritz F (2015). Comprehensive evaluation of electronic medical record system use and user satisfaction at five low-resource setting hospitals in Ethiopia. JMIR Med Inform.

[29] Kimaro H, Nhampossa J (2007). The challenges of sustainability of health information systems in developing countries: comparative case studies of Mozambique and Tanzania. J Health Inform Dev Ctries.

[30] Charmaz K, Denzin NK, Lincoln YS (2000). Grounded theory: objectivist and constructivist methods. Handbook of qualitative research.

[31] Were MC, Meslin EM. Ethics of implementing Electronic Health Records in developing countries: points to consider. In: AMIA Annual Symposium Proceedings: 2011. Washington DC: American Medical Informatics Association; 2011: 1499. PMC3243215 22195214

[32] Driessen J, Cioffi M, Alide N, Landis-Lewis Z, Gamadzi G, Gadabu OJ, Douglas G (2013). Modeling return on investment for an electronic medical record system in Lilongwe, Malawi. J Am Med Inform Assoc.

[33] Fritz F, Kebede M, Tilahun B (2014). The need for cost-benefit analyses of eHealth in low and middle-income countries. Stud Health Technol Inform.

