# Supplementary material for: Sustainability of health information systems: a three-country qualitative study in southern Africa
Source: BMC Health Serv Res. 2017 Jan 10;17:23. doi: 10.1186/s12913-016-1971-8 (PMC5223327; doi:10.1186/s12913-016-1971-8)
Supplement: Additional file 1: — Health information system sustainability interview questions. (DOCX 18 kb) [file 12913_2016_1971_MOESM1_ESM.docx]

**Health Information System Sustainability Interview Questions**

Thank you for your willingness to participate in an interview related to Health Information Systems (HIS) and HIS-related organizations in <country>. There are no right or wrong answers to these questions. Your name will not be linked in any public reports to any information that you individually provide. I will cover several different areas related to ___________________________ (health information system). This is not a test of your knowledge, if you do not know an answer to a specific question that is okay. After the interview we will ask to see some documents related to the system. Do you have any questions for us before we get started?

**Before beginning questions related to the system, I would like to ask you a few questions about your job**.

1. What is your job title?
2. How long have you been working as a ______________________ (job title)?
3. How long have you worked at this facility?
4. Have you previously worked in another health facility?

**Thank you. Let’s move on to questions specific to the health information system product now. I would like to start by asking you questions about some key characteristics of the program that developed and/or strengthened this product or system. The “product” in question is <name, description>.**

1. What are the specific goals of this product or HIS component?
   1. How are these goals monitored?
   2. Are updates/progress on meeting these goals reported to anyone?
      1. How often are these updates provided?
2. How were the goals of this program developed?
   1. Have these goals changed over time?
   2. Who participated in the creation of these goals?
3. Who participated in the design of this product?
   1. Were the persons utilizing this system on a daily basis consulted during the design?
   2. Have they been involved in modifications to the system?
4. How well was this project championed or advocated to stakeholders outside of project/program staff?
5. How long has the system been operating in the country?
   1. How long has this system been in place at this location?
6. Has the design of this project aligned with the country-level strategic plan for health systems?
7. Do you think experiences with the system will have an impact on the next cycle of strategic planning?
8. Does this system interact with any other health system currently or will it in the future?
9. Does a plan exist where donor reliance of financial support diminishes?
   1. Do you know what this plan is?
   2. How are you moving towards ownership of this system (decreased reliance on external financial and technical assistance)?

**I will now ask some general questions about the operation of the system.**

1. What would happen if this product or HIS component stopped operating?
   1. Is there a contingency plan in place if the system were to stop operating?
   2. How would the absence of this system impact your daily work?
2. Tell me about your experience using this system?
   1. What do you like about this system?
   2. What do you dislike about this system?
3. At how many sites is the system implemented?
4. Do sites have varying numbers of stations?
5. How has this system impacted this health facility?
   1. Who is responsible for this system at this health facility?
6. Are you able to modify the system at this location or district?
   1. How does this occur?
   2. What types of modifications can be made?

**Now, I would like to ask you some questions about system support. When I say support I mean technical support.**

1. Who provides technical support for this system?
2. Can you tell me what kind of non-financial support you receive?
   1. Have you worked at another health facility previously that also used this system?
   2. If yes: Did you receive the same level of support at that facility?
3. How many people are employed to do system support and maintenance?
4. Do you have support people/technicians that work in this district?
   1. What kinds of problems are they able to fix?
   2. Can you tell me where they work?
   3. If no, where are the support technicians/persons located?
5. Has the system ever needed to be fixed because it stopped working?
   1. How long did it take to repair the system?
6. How often are upgrades to the system made?
7. If changes to the system are needed, can you ask for these changes to be made?
   1. Do you know if anyone else at this facility can ask for changes to be made?
   2. How are these changes made locally?
      1. Are these changes reported to anyone?
8. Have you previously asked for changes to be made, either in how the system operates or by asking for enhancements to be made?
   1. How did you make this/these request(s)?
   2. Was/Were your request(s) successful?

**Now, I would like to ask you some financial questions about the system?**

1. Who provides financial support for this system?
   1. Has this changed throughout the life of the system?
2. Is there money to replace system hardware if it breaks or needs to be upgraded?
   1. Where does this money come from?
3. Is there a specific budget to upgrade system hardware equipment?
   1. Who maintains this budget?
4. How would you characterize interactions between donor organizations and the host organization?
   1. Does the host country also receive technical assistance from this/these donors?
5. Does the strategic plan include a costing model for the program or is one being worked on?

**Moving away from financial questions, I would now like to ask you some questions about staffing and system training.**

1. Has the system been expanded in the last year?
2. How many staff members actively utilize this system?
   1. Can you tell me more about the types of positions these people hold (i.e. doctor, nurse, technician, data entry clerk, etc.)?
3. Have staff been added/hired in the last year?
4. Have staff left their job in the last year?
5. What kind of training did you receive regarding how to operate this sytem?
   1. Who paid for this training?
6. Where did this training take place?
7. Since this initial training, have you received any follow up training?
   1. Where did this training take place?
   2. How often have trainings occurred?
   3. Do you know who pays for these training?
8. If you have a question regarding system operation, where do you go to find help?
9. Does the system have an online help feature that you can access?
10. Which organizations conduct these trainings?

**I would now like to ask you a few questions about how this system impacts you personally.**

1. Have you ever been asked how this system works for you?
   1. Who asked you this?
2. How has this system impacted your work both positively and negatively?
3. What parts of this system are essential to your work?
4. Overall, do you find this system useful?

**Thank you. We have reached the end of the interview. Do you have anything else you would like to share or any additional questions for me?**
